# Alterations of phenotype, physiology, and functional substances reveal the chilling-tolerant mechanism in two common *Olea Europaea* cultivars

**DOI:** 10.3389/fpls.2023.1046719

**Published:** 2023-02-01

**Authors:** Chenkai Jiang, Wenjun Hu, Hongling Lu, Lin Chen, Erli Niu, Shenlong Zhu, Guoxin Shen

**Affiliations:** ^1^ Institute of Sericulture and Tea, Zhejiang Academy of Agricultural Sciences, Hangzhou, Zhejiang, China; ^2^ Institute of Crops and Nuclear Technology Utilization, Zhejiang Academy of Agricultural Sciences, Hangzhou, Zhejiang, China

**Keywords:** amino acids, cold resistance, gene expression, lipids, terpenes

## Abstract

Olive suffers from cold damage when introduced to high-latitude regions from its native warm climes. Therefore, this study aims to improve the adaption of olive to climates in which it is cold for part of the year. The phenotype, physiological performance, nutrient content, and gene expression of olive leaves (from two widely planted cultivars) were examined after cultivation in normal and cold stress conditions. The results showed that the cold-tolerant cultivar possessed stronger photosynthesis efficiency and higher anti-oxidase activity after cold treatment than the cold-sensitive cultivar. Alteration of gene expression and metabolites in the amino acid metabolism, glycerolipid metabolism, diterpenoid biosynthesis, and oleuropein metabolism pathways played an important role in the cold responses of olive. Furthermore, the construction of the network of genes for ubiquitination and metabolites suggested that polyubiquitination contributes most to the stable physiology of olive under cold stress. Altogether, the results of this study can play an important role in helping us to understand the cold hardiness of olive and screen cold-resistant varieties for excellent quality and yield.

## Introduction

Olive (*Olea europaea* L.) is one of four woody oil plants in the world. Cultivated olive was first founded in Asia Minor and then spread to Mediterranean regions, such as Greece, Italy, and Spain (Wang et al., 2022). Oil extracted from olive fruits, known as liquid gold, contains abundant unsaturated fatty acids, which provide numerous health benefits (Wang et al., 2022). The olive industrial system and the international olive market are consistently expanding, especially in China. Olive has been cultivated in China on a large scale since the 1960s due to its high quality and the economic value of its oil (Hernández-García et al., 2021). The optimum temperature for olive growth is 20–30°C (Wang et al., 2018). Olive trees cannot survive below −12°C (Gomez Del Campo and Barranco, 2005). China is located in East Asia on the west shore of the Pacific and has a monsoon climate with hot summers and cold winters. Chilling and frost stress during winter and spring in China restrict olive growth and threaten its normal physiological function. Surprisingly, olive trees cultivated under cold climates produce unexpected benefits, as cold improves olive fruit quality by slowing down the post-maturation process (Chialva et al., 2021). Therefore, it is important to breed and domesticate cold-resistant olive varieties.

When breeding cold-tolerant olive accessions, it is important to understand the chilling tolerance principle. Several studies have reported the complex mechanism underlying cold tolerance in plants, which mainly involves physiological alterations, gene regulation, and antioxidant enzymes (Eom et al., 2022). The expression level of HDG1 was associated with cold tolerance, and the LTR inserted in the first intron of HDG1 might be cold-inducible in winter rapeseed (Wu et al., 2022). Plants trigger a series of protective reactions regulated by these related genes under low-temperature stress. The plant membrane system contains proteins that receive, transduce, and cascade external temperature signals. Plants regulate cytosol concentration by relying on osmotic regulators, such as soluble sugars and proteins, to maintain normal cell morphology. Osmotic stress is the dominant factor affecting the capacity of winter wheat varieties to survive at low temperatures (Bao et al., 2022). The hormonal signal network integrates external information into the endogenous system and activates stress response pathways, thus bringing about cold resistance (Marina et al., 2016). The antioxidant enzyme system concerning superoxide dismutase (SOD), catalase (CAT), peroxidase (POD), and ascorbate peroxidase (APX) eliminates excess reactive oxygen species induced by cold stress (Baier et al., 2019). *Zanthoxylum bungeanum* adapted to cold stress by altering signal transduction, plant hormones, transcription factors, protein modification, functional proteins, and other physiological indexes (Tian et al., 2021). In conclusion, it is viable to introduce and acclimatize plants by selecting cold-tolerant genotypes integrated with cultivation practices based on cold resistance mechanisms.

The distinct climatic and environmental differences between native regions and China are the greatest challenge for olive introduction. Only 28 out of 165 olive cultivars that have been introduced from the Mediterranean are able to satisfactorily produce fruit in China (Wang et al., 2019). However, there has been a lack of comprehensive studies on the phenotype, physiology, functional substances, and gene expression of olive tree exposed to low temperatures. Therefore, two widely cultivate oil varieties, Arbequina and Koroneiki, were planted under normal and cold stress conditions in the present study. Representative genes and nutrients related to cold resistance were identified and used to construct regulatory networks. This process shed light on the mechanism underlying cold hardiness and could improve the breeding and selection of cold-tolerant olive varieties *via* primary nutrients in the future.

## Materials and methods

### Plant materials

Arbequina and Koroneiki are native to Spain and have been cultivated on a large scale in Gansu, Yunnan, and Sichuan in China. They are excellent olive cultivars that have been successfully bred for more than 20 years since their introduction and after regional experiments according to systematic breeding procedures. The identification numbers of Arbequina and Koroneiki are ‘National R-ETS-OE-005-2018’ and ‘National R-ETS-OE-004-2018’, respectively. Two 1-year-old olive cultivars, Arbequina and Koroneiki, were planted normally in a controlled environmental growth chamber with 60% relative humidity, a light intensity of 12,000 lux, and 16 h light and 8 h dark at 28°C. A total of 24 healthy seedlings were prepared for subsequent treatments. Six seedlings of each cultivar were used for each treatment.

### Cold treatment

Olive plants with the same growth vigor were moved into an artificial climate chamber. The temperature was lowered to 0°C at a rate of 2°C per hour and maintained at 0°C for 24 h. The control groups were cultivated at 28°C. The second to fourth leaves under the terminal bud were plucked and flash frozen in liquid nitrogen and stored at −80°C for transcriptome and metabolome analysis. Samples for phenotypical observation and physiological and biochemical analysis were harvested after recovery for 24 h. Samples were gathered from at least three plants.

### Physiological detection

The second leaf of the olive plants was cut into 2-mm strips and shaken in distilled water for 30 min. The electrical conductivity of the leaves was measured, with the sample marked as CA and the distilled water blank control marked as blank1. After immersion in boiling water for 30 min, a second electrical conductivity measurement was taken, with the sample marked as CB and the distilled water marked as blank2. Relative electrolyte leakage = $\frac{\text{CA}-\text{blank}1}{\text{CB}-\text{blank}2}$ ×100% (Rao et al., 2021). SOD and APX activity were evaluated using an Elisa kit (Shanghai Enzyme-linked Biotechnology Co., Ltd., China). CAT activity was evaluated using an ELISA kit (Shanghai Zhenke Biotechnology Co., Ltd., China). POD activity was evaluated using a kit (Jieshikang Biotechnology Co., Ltd., China). The content of proline and MDA was measured using a kit (Nanjing Jiancheng Biotechnology Co., Ltd., China). H_2_O_2_ content was evaluated using an H_2_O_2_ content assay kit (Beijing Boxbio Science & Technology Co., Ltd., China). The maximal PS II efficiency of leaves was measured after 12 h dark treatment using a Mini-Pam photosynthesis yield analyzer (WALZ, German). Gibberellin (GA) content was absolute quantified using UPLC (Waters, USA). The dried leaves were broken into powder. A total of 30 ml of boiled water was added to 0.1 g of power and boiled for 30 min. An aliquot of 1 ml of supernatant fluid after filtering was added to 4 ml of anthrone. Soluble sugar was measured according to the light absorption value at 620 nm, with distilled water used as a blank control. The homogenate was obtained from 0.3 g of fresh leaves broken up in 5 ml of distilled water. Coomassie brilliant blue G-250 (5 ml) was added into 0.1 ml of the clean fluid after filtering. Soluble protein was measured according to the light absorption value at 295 nm, with distilled water used as a blank control.

### RNA extraction, library preparation, and Illumina Hiseq sequencing

Total RNA was extracted from the leaves using an RNAprep Plant kit (Tiangen Biotech Co., Ltd., China) according to the manufacturer’s instructions. Then, RNA quality was determined using a NanoDrop spectrophotometer (Thermo Fisher Scientific, USA). High-quality RNA sample (OD260/280, 1.9–2.1; OD260/230, ≥2.0) was used to construct a sequencing library. RNA-seq transcriptome libraries were prepared using an Illumina TruSeqTM RNA sample preparation Kit (San Diego, CA, USA). The 100–200-bp libraries were used to select cDNA target fragments on 2% ultra-agarose, which were subsequently amplified by PCR. Afterwards, paired-end libraries were sequenced using an Illumina NovaSeq 6000 (Shanghai Biozeron Co., Ltd., China).

### Read quality control and mapping

The raw paired-end reads were trimmed and quality controlled using Trimmomatic (http://www.usadellab.org/cms/uploads/supplementary/Trimmomatic) with the following parameters: SLIDINGWINDOW = 4:15 and MINLEN = 75. Then, clean reads were separately aligned to the *Olea europaea* cv. ‘Arbequina’ reference genome (https://ngdc.cncb.ac.cn/gwh/Assembly/10300/show) using orientation mode and hisat2 software with default parameters. The quality assessment of these data was carried out using qualimap_v2.2.1. Htseq was used to count each gene read.

### Differential expression analysis and functional enrichment

The expression level of genes was calculated using the fragments per kilobase of exon per million mapped reads (FRKM) method. The R statistical package edgeR was used for differential expression analysis. The differential expression genes (DEGs) between two samples were selected based on the following criteria: logarithmic fold change of ≥2 and a false discovery rate of ≤0.05. The functions of the DEGs were annotated by GO functional enrichment and KEGG pathway analysis with Goatools and KOBAS. DEGs that had a Bonferroni-corrected P-value ≤0.05 were significantly enriched in GO terms and metabolic pathways.

### Quantitative reverse-transcription PCR (qRT-PCR) analyses

Twelve genes responsible for the metabolism of lipids, terpenes, and ubiquitin were randomly selected for the validation of gene expression using qRT-PCR (**Figure S2**). The primers used are listed in **Table S1**. The qRT-PCR reactions were conducted using the following parameters: 95°C for 10 min, 45 cycles at 94°C for 10 s, and 58°C for 15 s. Three independent biological replicates of each reaction were obtained, with GAPDH used as a reference gene. Fluorescence intensity was measured using a LightCycler 480 machine (Roche, Sussex, UK), and the relative expression values of genes were calculated using the 2^-ΔΔCt^ method.

### Sample extraction and LC-MS conditions

A 10-ml volume of 70% methanol was added to 0.2 g of leaf powder. The mixture was extracted at 35°C for 45 min. After centrifugation, the supernatants were filtered through a 0.22-μm filter membrane. The extraction was stored at −80°C until detection. Quality control (QC) was mixed from 100 μl of the extract of all samples.

Metabolites of samples were detected using a Vanquish UPLC (Thermo Scientific, USA)-Q-Orbitrap (Thermo Scientific, USA) with a Hypesil GOLD column ((100×2.1 mm, 1.9 μm). Solvent A consisted of water containing 0.1% formic acid (Thermo, LC), and solvent B was acetonitrile (Thermo, LC). The injection volume was 5 μL. The flow rate and column temperature were 0.4 mL/min and 40°C, respectively. QC samples were inserted across every five samples.

### Metabolic data processing

The total ion flow graph was generated in positive and negative ion modes. The original data obtained by mass spectrometry were processed in Compound Discoverer 2.1, including peak extraction, peak alignment, and peak correction. Principal component analysis (PCA) and partial least squares discriminant analysis (PLS-DA) based on contents of metabolites was performed and visualized using ggplot2. Differentially accumulated metabolites (DAMs) were analyzed using DESeq2 according to a fold change of >2 and P-<0.05. These DAMs were aligned to KEGG pathways (https://www.kegg.jp/). Volcano plots of DAMs were plotted in ggplot2. Pearson correlation coefficient (PCC) values between metabolite and metabolite, gene and gene, and metabolite and gene were calculated in R 4.1.2. The network of genes and metabolites was visualized based on the PCC in Cytoscape 3.9.1.

## Results

### Phenotypic and physiological responses of olive plants under cold stress

**Figure 1:**
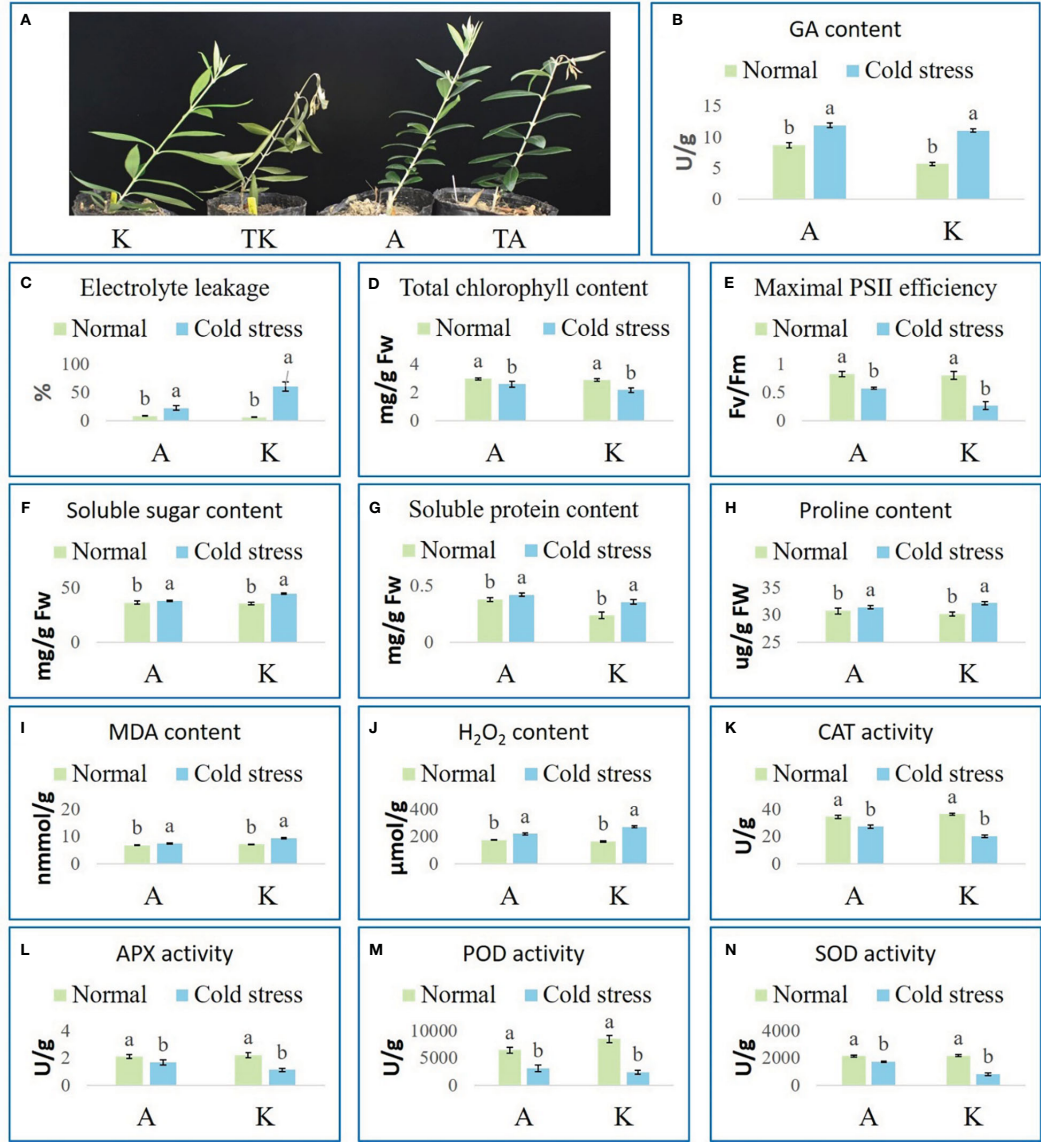
Phenotypes and physiological indexes of olive plants under normal and cold conditions. **(A)** Phenotypes. **(B)** GA content. **(C)** Electrolyte leakage. **(D)** Total chlorophyll content. **(E)** Maximal PSII efficiency. **(F)** Soluble sugar content. **(G)** Soluble protein content. **(H)** Proline content. **(I)** MDA content. **(J)** H_2_O_2_ content. **(K)** CAT activity. **(L)** APX activity. **(M)** POD activity. **(N)** SOD activity. ‘A’ and ‘K’ represent Arbequina and Koroneiki under normal conditions; ‘TA’ and ‘TK’ represent Arbequina and Koroneiki under cold conditions; ‘a’ and ‘b’ represent significant difference based on least significant difference (t-test).

Two domestic varieties, Arbequina and Koroneiki, were cultivated under normal (A and K, respectively) and cold stress conditions (TA and TK, respectively) and their performance was observed. TK lost vitality and had curled and yellow leaves, and their fresh shoots completely drooped (**Figure 1A**). The fresh shoots of TA bent; none of the other parts had noticeable cold damage (**Figure 1A**). To further explore the cold resistance of the two varieties, electrolyte leakage, photosynthetic performance, osmotic adjustment substances, and the activity of major antioxidant enzymes were examined. After cold stress, GA content, relative electrolyte leakage, soluble sugar content, protein, proline, malondialdehyde (MDA), and H_2_O_2_ increased in both varieties (**Figures 1B, C, F, G–J**). Simultaneously, chlorophyll content, maximal PS II efficiency, and catalase (CAT), ascorbate peroxidase (APX), peroxidase (POD), and superoxide dismutase (SOD) activity decreased in both varieties (**Figures 1D, E, K–N**). Additionally, TA had a lower level of MDA and H_2_O_2_, stronger photosynthesis efficiency, and higher CAT, APX, POD, and SOD activity than TK. These results indicate that Arbequina is more cold resistant than Koroneiki.

### Functional analysis of DEGs of olive plants under cold stress

**Figure 2:**
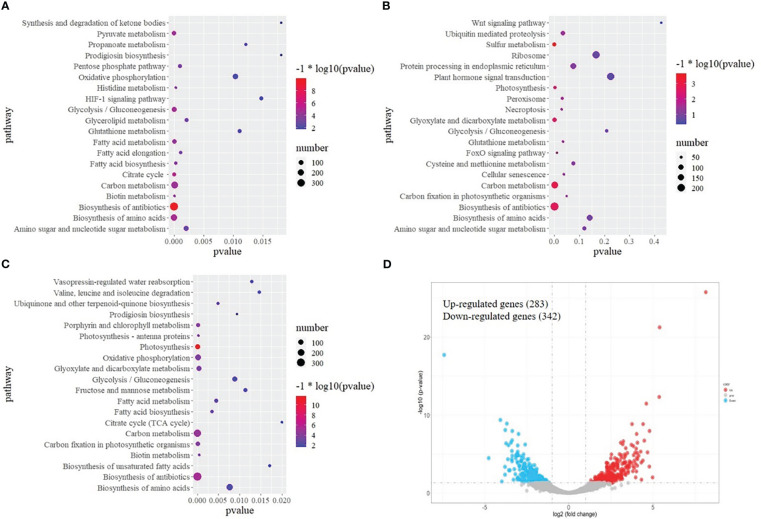
KEGG pathway enrichment of DEGs. **(A)** Pathway enrichment based on DEGs between normal and cold-treated Arbequina. **(B)** Pathway enrichment based on DEGs between normal and cold-treated Koroneiki. **(C)** Pathway enrichment based on DEGs between cold-treated Arbequina and Koroneiki. **(D)** Volcano plots of DEGs between cold-treated Arbequina and Koroneiki.

To explore the molecular events of olive plants under cold stress, transcriptomic analysis of leaves was performed. After removing low-quality reads, a total of 627,324,150 clean reads were obtained. The percentage of Q30 was 89.45–93.71%, suggesting the transcriptome sequencing data was of high quality. A total of 52,268 genes were functionally annotated in the databases. The biological functions of DEGs between TA and A, TK and K, and TA and TK were further assessed. **Figure 2A** shows that the top 5 KEGG pathways significantly enriched by DEGs between TA and A were ‘biosynthesis of antibiotics’, ‘biosynthesis of amino acids’, ‘glycolysis/gluconeogenesis’, ‘carbon metabolism’, and ‘fatty acid biosynthesis’. The top 5 KEGG pathways significantly enriched by DEGs between TK and K were ‘biosynthesis of antibiotics’, ‘plant hormone signal transduction’, ‘carbon metabolism’, ‘biosynthesis of amino acids’, and ‘ubiquitin-mediated proteolysis’ (**Figure 2B**). The top 5 KEGG pathways significantly enriched by DEGs between TA and TK were ‘carbon metabolism’, ‘biosynthesis of antibiotics’, ‘biosynthesis of amino acids’, ‘oxidative phosphorylation’, and ‘photosynthesis’ (**Figure 2C**). Furthermore, ‘biosynthesis of amino acids’, ‘biosynthesis of antibiotics’, and ‘glycolysis/gluconeogenesis’ were the most common top 20 significantly enriched pathways in the three above-mentioned comparative groups. After cold stress, 283 and 342 genes were upregulated and downregulated, respectively, in TA compared with TK (**Figure 2D**).

### Overview of olive plant metabolites under cold stress

**Figure 3:**
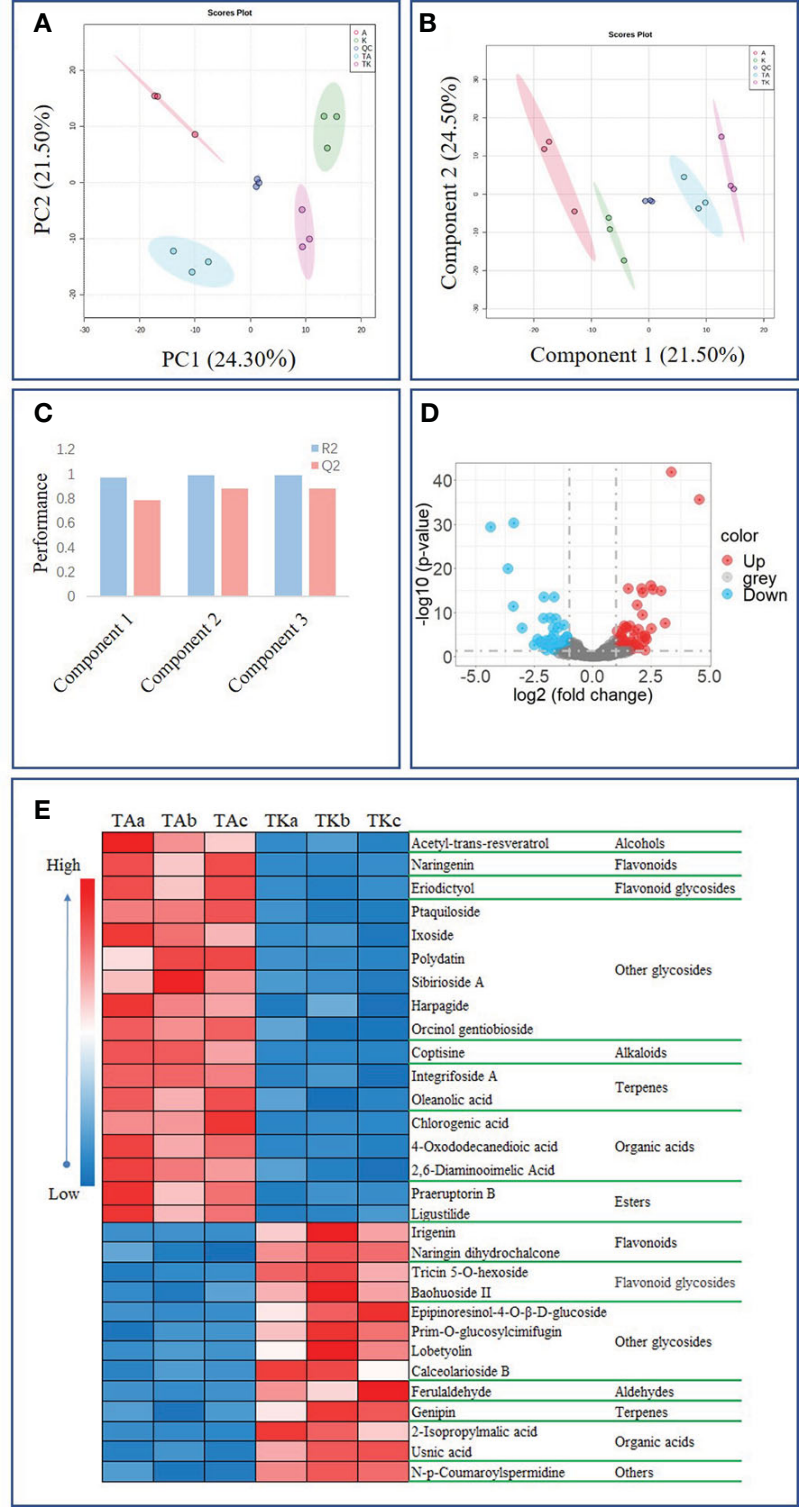
Metabolome analysis of olive plants. **(A)** PCA plots of metabolites in olive plants. **(B)** PLS-DA plots of metabolites in olive plants. **(C)** Tenfold cross-validation of PLS-DA. **(D)** Volcano plots of DAMs between TA and TK. **(E)** Heat map of the top 30 DAMs between TA and TK. ‘TA’ and ‘TK’ represent Arbequina and Koroneiki under cold conditions.

Analysis of the non-targeted metabolome was performed using an LC-MS/MS system. A total of 971 features were detected, 573 of which were identified (**Table S2**). These metabolites could be classified into 18 categories. As described in **Figure S1**, organic acids, glycosides, and esters were the top three classifications. Most of the glycosides (117), were flavonoid glycosides (33). Afterwards, an unsupervised PCA and a supervised PLS-DA were applied to all samples. The score graph (**Figure 3A**) shows the QC samples distributed at the center, whereas other samples were scattered around the QC samples and divided into four groups. The result of PCA shows that PC1 and PC2 accounted for 24.30% and 21.50% of the variation rate, respectively (**Figure 3A**). Subsequently, a supervised PLS-DA was applied to further investigate the effects of cold stress on metabolites. **Figure 3B** shows obvious metabolic variations induced by cold stress in Arbequina and Koroneiki leaves. The calculated R^2^ and Q^2^ were up to 0.99 and 0.88 according to 10-fold cross-validation of the first three components (**Figure 3C**), indicating that the result from the PLS-DA approach was reliable.

There were 56 upregulated and 46 downregulated DAMs in the cold-treated leaves of Arbequina and Koroneiki (**Figure 3D**). Among them, 30 DAMs were metabolic markers that had a log2 fold change of >1 and P<0.01 and included 10 glycosides, five organic acids, three flavonoids, three flavonoid glycosides, three terpenes, two esters, one alcohol, one aldehyde, one alkaloid, and one unclassified compound (**Figure 3D**). The content of chlorogenic acid and coptisine in TA was 23.60, 10.29 times that of TK (**Figure 3E**), while the content of epipinoresinol-4-O-β-D-glucoside and ferulaldehyde in TA was 0.05 and 0.08, respectively, that of TK (**Figure 3E**).

### Primary metabolism is affected by cold stress

**Figure 4:**
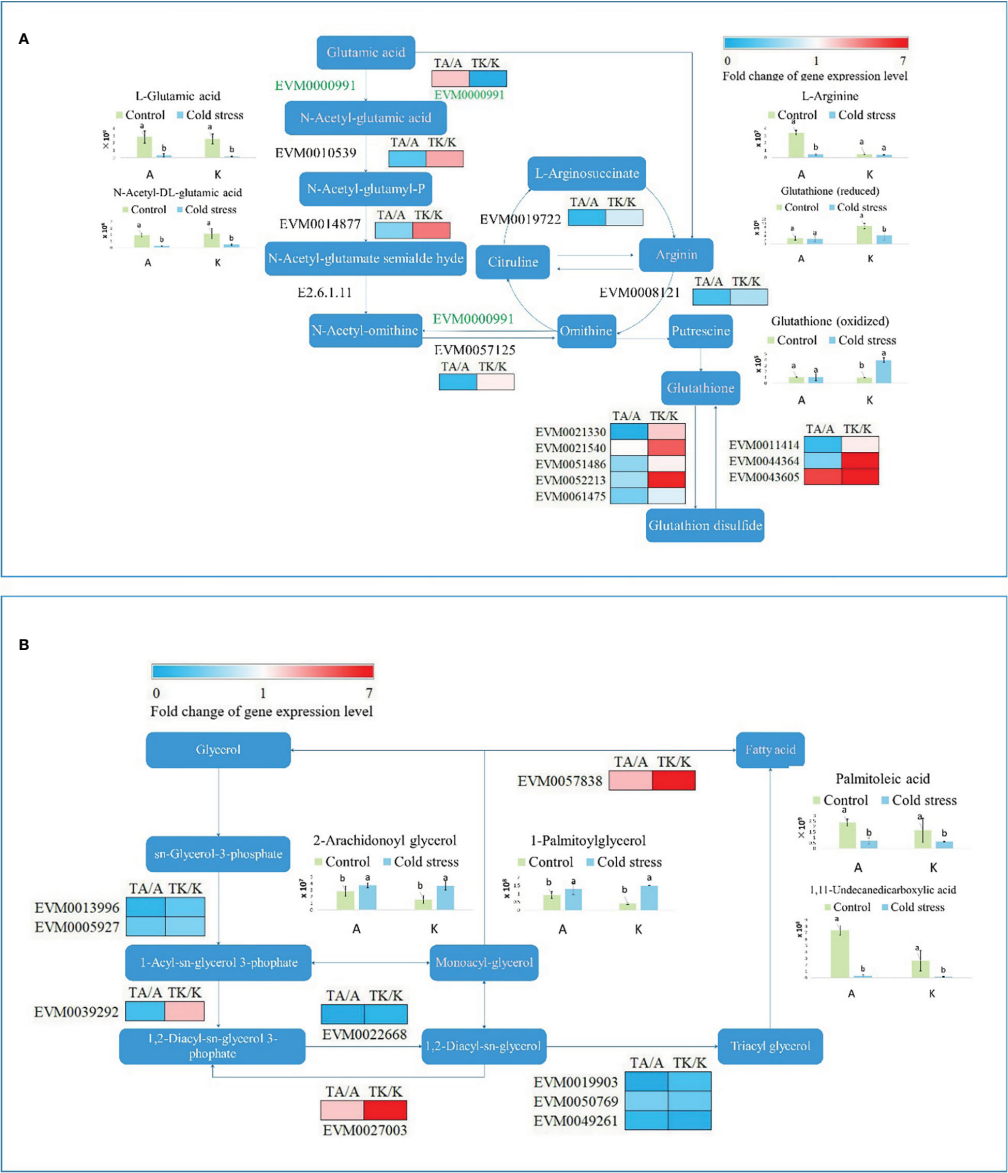
The primary metabolism pathway influenced by cold stress. **(A)** The amino acid biosynthesis pathway. **(B)** The glycerolipid metabolism pathway. ‘a’ and ‘b’ represent significant difference based on least significant difference (t-test). ‘A’ and ‘K’ represent Arbequina and Koroneiki under normal conditions; ‘TA’ and ‘TK’ represent Arbequina and Koroneiki under cold conditions.

As previously mentioned, the ‘biosynthesis of amino acids’ and ‘glycolysis/gluconeogenesis’ pathways were dramatically influenced by cold stress in both Arbequina and Koroneiki. After cold treatment, EVM0010539, EVM0014877, EVM0057125, EVM0021330, EVM0021540, EVM0051486, EVM0052213, EVM0011414, and EVM0044364 were downregulated in Arbequina, whereas they were upregulated in Koroneiki (**Figure 4A**). EVM0019722, EVM0008121, and EVM0061475 were downregulated in both varieties (**Figure 4A**). Only EVM0043605 was upregulated in both varieties (**Figure 4A**). The accumulation of L-glutamic acid and N-acetyl-DL-glutamic acid was restricted by cold stress in both varieties (**Figure 4A**). The content of L-arginine obviously reduced in TA but not in TK (**Figure 4A**). Reduced and oxidized glutathione maintained equilibrium in TA (**Figure 4A**). However, a decrease in reduced glutathione accompanied an increase in oxidized glutathione in TK (**Figure 4A**). EVM0021330, EVM0021540, EVM0051486, EVM0052213, and EVM0061475 regulated the reaction from glutathione to glutathione disulfide. Their expression levels decreased in TA. This shows that glutathione generation is enhanced, and the dynamic equilibrium of different glutathione types contributes to cold tolerance.

Besides amino acid biosynthesis, fatty acid metabolism is another primary metabolism associated with cold response in olive. As shown in **Figure 4B**, EVM0013996, EVM0005927, EVM0022668, EVM0019903, EVM0050769, and EVM0049261 were downregulated in the lipid metabolism pathway in both varieties after chilling stress. By comparison, EVM0027003 and EVM0057838 were upregulated in both varieties after low-temperature stimulation, and their expression levels increased more in TK (**Figure 4B**). EVM0039292 was downregulated in TA but upregulated in TK (**Figure 4B**). The content of 2-arachidonoyl glycerol respectively rose to 1.31 and 2.35 times in Arbequina and Koroneik in response to cold stress (**Figure 4B**). The content of 1-palmitoylglycerol respectively rose to 1.40 and 3.72 times in Arbequina and Koroneiki in response to cold stress (**Figure 4B**). Interestingly, the content of two unsaturated fatty acids (palmitoleic acid and 1,11-undecanedicarboxylic acid) declined after cold treatment in both varieties (**Figure 4B**). In detail, palmitoleic acid content decreased by 70.71% and 61.12% after cold treatment in Arbequina and Koroneiki, respectively, and 1,11-undecanedicarboxylic acid content dropped by 95.62% and 94.14% after cold treatment in *arbequina* and *koroneik*, respectively. It seems that fatty acid reserves rather than its transformation play an important role in cold tolerance in olive.

### Terpene metabolism is enhanced by cold stress

**Figure 5:**
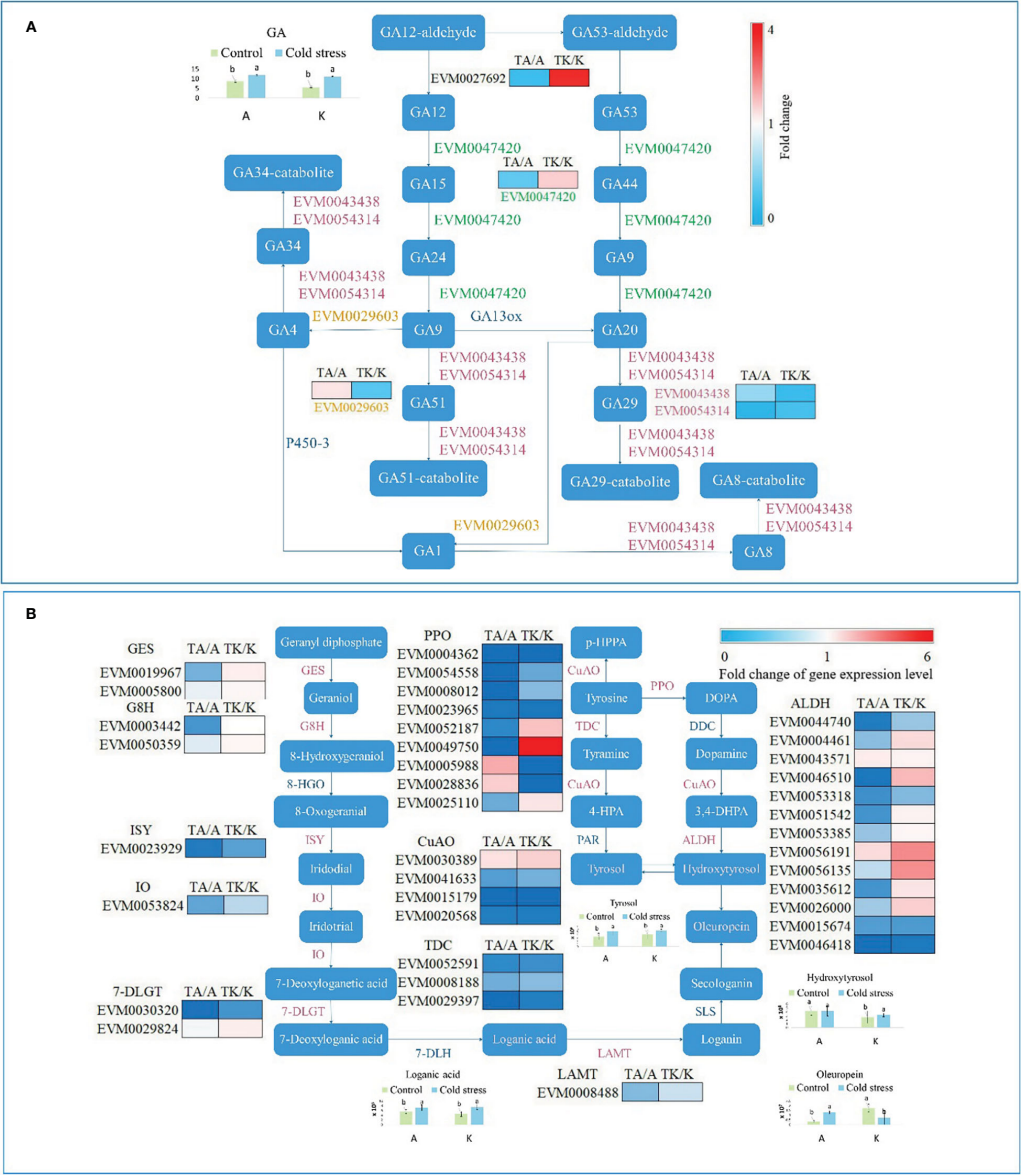
The terpene metabolism pathway affected by cold stress. **(A)** The diterpenoid biosynthesis pathway. **(B)** The oleuropein biosynthesis pathway. GES, geraniol synthase; G8H, geraniol 8-hydroxylase; 8-HGO, 8-hydroxygeraniol oxidoreductase; ISY, iridoid synthase; IO, iridoid oxidase; 7-DLGT, 7-deoxyloganetic acid-O-glucosyl transferase; 7-DLH, 7-deoxyloganic acid hydroxylase; LAMT, loganic acid methyltransferase; DDC, DOPA decarboxylase; SLS, secologanin synthase; PPO, polyphenol oxidase; TDC, tyrosine decarboxylase; CuAO, primary amine oxidase; PAR, phenylacetaldehyde reductase; ALDH, alcohol dehydrogenase. ‘A’ and ‘K’ represent Arbequina and Koroneiki under normal conditions; ‘TA’ and ‘TK’ represent Arbequina and Koroneiki under cold conditions; ‘a’ and ‘b’ represent significant difference based on least significant difference (t-test).

According to hormone determination, GA content increased after cold stimulation. GA, a diterpenoid, regulates the growth and development of plants and integrates with other hormones. In the current study, five genes mapped to the diterpenoid biosynthesis pathway (**Figure 5A**). Under cold stress, EVM0043438 and EVM0054314 were downregulated in Arbequina (cold-tolerant cultivar) and Koroneiki (cold-sensitive cultivar) (**Figure 5A**). EVM0029603 was upregulated in TA, whereas EVM0027692 and EVM0047420 were downregulated (**Figure 5A**). EVM0029603, EVM0027692, and EVM0047420 presented the contrary responses in TK (**Figure 5A**).

Oleuropein, composed of hydroxytyrosol, elenolic acid, and glucoside moieties, is synthesized through the iridoid biosynthesis pathway (Zheng et al., 2021). It is the main bioactive polyphenolic compound in olive leaf, fruit, root, and branch (Zheng et al., 2021). Genes and metabolites in the oleuropein biosynthesis pathway significantly responded to cold stimulation, as shown in **Figure 5B**. GES (geraniol synthase), G8H (geraniol 8-hydroxylase), ISY (iridoid synthase), IO (iridoid oxidase), 7-DLGT (7-deoxyloganetic acid-O-glucosyl transferase), TDC (tyrosine decarboxylase), LAMT (loganic acid), and the majority of PPO (polyphenol oxidase), CuAO (primary amine [copper-containing] oxidase), and ALDH (alcohol dehydrogenase) were inhibited in TA (**Figure 5B**). However, the expression of these genes decreased less or even increased in TK (**Figure 5B**). The content of oleuropein increased in Arbequina after cold stress but decreased in Koroneiki (**Figure 5B**). Although Koroneiki contained more oleuropein than Arbequina under normal conditions, Arbequina accumulated more oleuropein under cold conditions (**Figure 5B**). The biosynthesis of loganic acid and tyrosol was enhanced under cold stress in both varieties (**Figure 5B**). The regulatory distinction in the oleuropein biosynthesis pathway between the two cold-treated varieties reveals that oleuropein metabolism is involved in the cold stress response in olive, and abundant oleuropein accumulation is helpful for resisting chilling stress.

### Ubiquitination promotes apoptosis after cold stress

**Figure 6:**
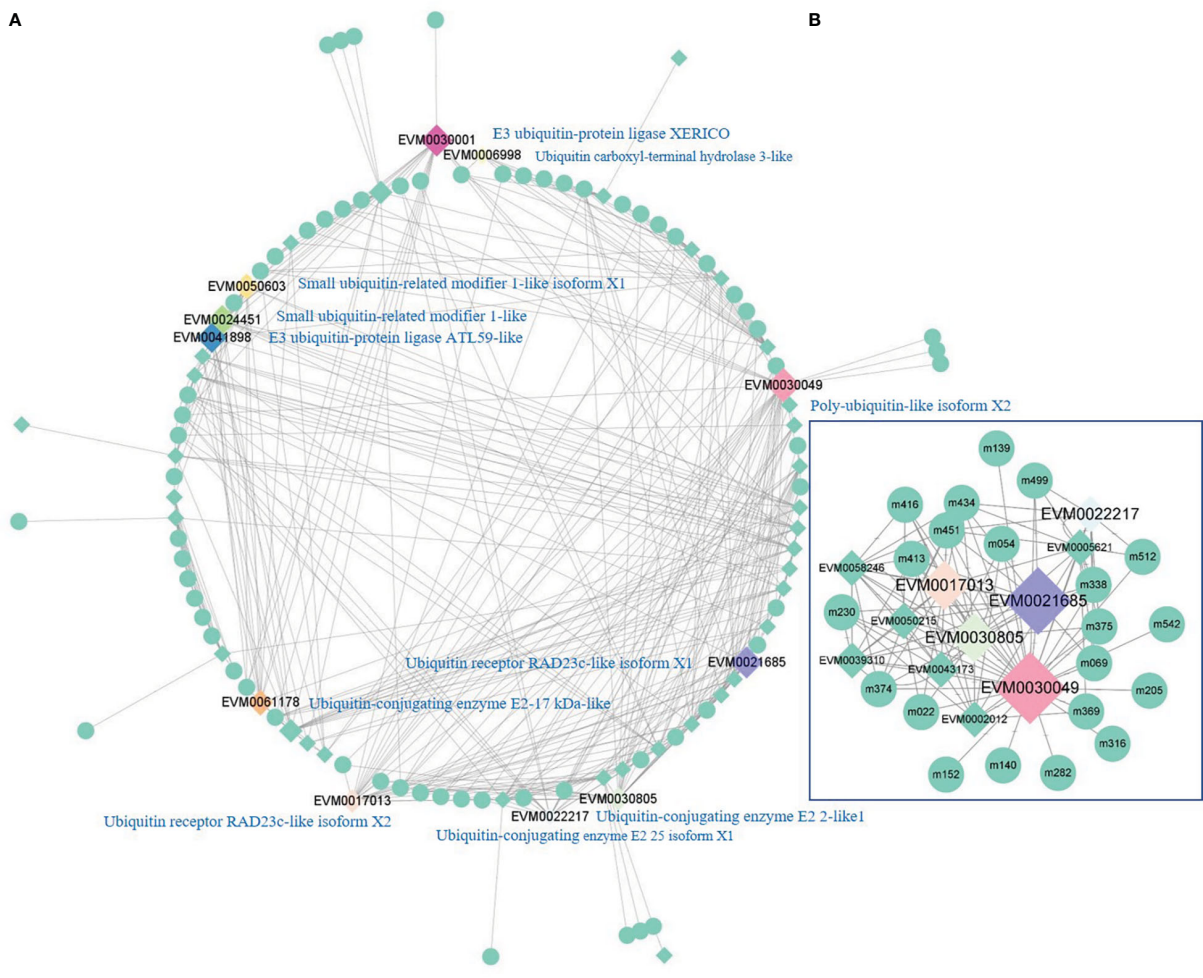
The network of genes for ubiquitination and metabolites with a Pearson correlation coefficient of >0.9. **(A)** Regulatory network of ubiquitination among genes and metabolites. **(B)** Regulatory network with polyubiquitin-like isoform X2 (EVM0030049) as the hub-gene. m022, 12-oxo phytodienoic acid; m054, dihydrojasmonic acid; m069, ganoderic acid A; m139, picrotoxinin; m140, monoolein; m152, 4-demethylepipodophyllotoxin; m205, naringin dihydrochalcone; m230, quercetin-3,4’-O-di-beta-glucopyranoside; m282, schizandrol A; m316, momordin Ic; m338, verbenalin; m369, L-glutamic acid; m374, N-acetyl-DL-glutamic acid; m375, L-homoserine; m413, methylchrysoeriol C-hexoside; m416, anwulignan; m434, cucurbitacin I; m451, sinapaldehyde glucoside; m499, luotonin A; m512, pantothenic acid; m542, 1-palmitoylglycerol.).

Ubiquitination is essential for cell apoptosis to clean up senescent and damaged cells. Therefore, it helps plants to maintain normal cell function under environmental stress. A total of 881 genes responsible for ubiquitination were identified in the current study. The PCC between these genes and metabolites was calculated. Among them, 42 genes were closely related to 66 metabolites, including 393 pairs of correlations with a PCC of >0.9. The relationships between these genes and metabolites are shown in **Figure 6A**. Eleven genes were closely related to more than five other genes or 12 metabolites. They encoded polyubiquitin (EVM0030049), ubiquitin-conjugating enzyme (EVM0030805, EVM0022217, and EVM0061178), ubiquitin receptor (EVM0021685 and EVM0017013), E3 ubiquitin-protein ligase (EVM0041898 and EVM0030001), small ubiquitin-related modifier (EVM002445 and EVM0050603), and ubiquitin carboxyl-terminal hydrolase (EVM0006998). Furthermore, EVM0030049 (polyubiquitin-like isoform X2) was closely related to 10 ubiquitination genes and 21 metabolites (**Figure 6B**). It constructed the biggest subnetwork, containing 119 pairs of correlations. Most of these metabolites were organic acids (12-oxo phytodienoic acid, dihydrojasmonic acid, ganoderic acid A, and pantothenic acid) and esters (picrotoxinin, monoolein, 4-demethylepipodophyllotoxin, 4-demethylepipodophyllotoxin, and 1-palmitoylglycerol). This demonstrates that among the various types of ubiquitination, polyubiquitination contributes most to stabilizing the physiology of olive under cold stress.

## Discussion

Two olive varieties presented distinct corresponding physiological performances after cold treatment. The imbalance between the generation and suppression of reactive oxygen species (ROS) causes oxidative stress. ROS elimination by antioxidants is a common response to abiotic stresses in plants. H_2_O_2_ is one of the most stable ROS. Catalase could convert H_2_O_2_ to H_2_O and O_2_ (Wang and Chu, 2020), thus protecting cells from the damaging effect of H_2_O_2_. The reduction of CAT activity led to more H_2_O_2_ being left over after cold stress. Similarly, the activity of major antioxidants SOD, POD, and APX was suppressed, and cold stress affected Arbequina less than Koroneiki. This may suggest that cold treatment at 4°C is a severe stress to olive plants. Since the provenance of olive is the warm Mediterranean regions, it is necessary to breed high cold-resistant cultivars for regions with cold winters. As for glutathione, one of the non-enzymatic antioxidants that assist with the elimination of ROS, it serves as an electron donor for glutathione peroxidase, which reduces hydrogen peroxide to water (Rattanawong et al., 2021). By donating an electron, glutathione is oxidized to glutathione disulfide. After cold stress, the expression level of genes encoding enzymes that catalyze the glutathione-to-glutathione disulfide process decreased in Arbequina. Simultaneously, the amount of reduced glutathione was not changed. It indicates that it is important to product sufficient glutathione in time for its steady number.

The physiological reactions are regulatedby transcriptionand metabolite level. It could discover the genes responsible for cold resistance *via* comparing the difference of gene expression patterns. After cold stress, 283 and 342 genes were upregulated and downregulated in cold-resistant Arbequina compared with cold-sensitive Koroneiki, respectively. Cold stress influenced amino acid biosynthesis (primary metabolism pathway) in olive (**Figure 4A**). Additionally, it demonstrated that glutathione generation was improved and showed once again that the equilibrium of reduced and oxidized glutathione is essential for cold tolerance in olive.

In addition to amino acid metabolism, lipid metabolism is another primary metabolic pathway significantly impacted by cold treatment. Numerous studies have reported that unsaturated fatty acids are closely associated with chilling responses. Under low-temperature conditions, cold-tolerant varieties have higher membrane lipid unsaturation and unsaturated fatty acid content, enabling the plant to maintain a certain degree of stability and fluidity of the membrane system (Liu et al., 2022). For instance, 3-day cold or dark treatment significantly induces eicosapentaenoic acid biosynthesis (Chua et al., 2020). Likewise, the cold-tolerant olive cultivar accumulated more unsaturated fatty acids (**Figure 4B**). The content of unsaturated fatty acids decreased under cold stress. Accordingly, we propose a hypothesis that fatty acid reserves, particularly unsaturated lipids, greatly contribute to cold tolerance and warrant further research. Thus, we will pay attention to the relationship between original lipid accumulation and cold resistance in subsequent investigations.

Both primary and secondary metabolites were influenced by plant hormones that possess complex and multiple functions for growth and development. GA is instrumental in the response to different environmental stimuli, such as temperature (Hernández-García et al., 2021). EVM0043438, EVM0054314, EVM0029603, EVM0027692, and EVM0047420 were identified in the diterpenoid biosynthesis pathway (**Figure 5A**). GA content increased under cold stimulation (**Figure 5A**). However, there is no regular variation law of GA in plants affected by low temperature. GA3 treatment effectively reduced the chilling injury index in mature green tomato fruit during long-term cold storage (Zhu et al., 2016). GA3 content in all tomato plants decreased after chilling stress (Wang et al., 2022), which was unexpected. Therefore, it is important to maintain GA at its appropriate level in plants to resist chilling injury. Additionally, it is important to maintain an appropriate GA composition, as different kinds of GAs show inconsistent effects in various plants (Zhang et al., 2021a). GA1, GA3, GA4, and GA7 are thought to function as bioactive hormones in plants (Hedden and Sponsel, 2015). The GA composition in olive leaves was not determined in this study and warrants further analysis so that the variation of the regulatory network under cold stress can be revealed. Besides GA, the secondary metabolite oleuropein is a terpenoid derivative with a metabolism similar to iridoids in *Fraxinus excelsior* and *Syringa josikaea* (Damtoft et al., 1993). The diversity of terpene compounds changes in response to environmental stimuli, offering plants protection against various biotic and abiotic stresses (Zhang et al., 2021b). Oleuropein biosynthesis is stimulated under cold stress (Ortega-García and Peragón, 2009). In the present study, oleuropein accumulated in cold-resistant olive plants and reduced in cold-sensitive plants, inferring that it plays a positive role in cold tolerance. Additionally, 38 DEGs were identified in the oleuropein biosynthesis pathway, including genes encoding GES, G8H, ISY, IO, 7-DLGT, TDC, LAMT, PPO, CuAO, and ALDH. A total of 202 genes have been identified in the oleuropein biosynthesis pathway to date (Rao et al., 2021). Nevertheless, the process of oleuropein generation from hydroxytyrosol and secologanin in still unclear, as are the genes responsible for oleuropein metabolism regulated by low temperature. Consequently, further clarification regarding oleuropein biosynthesis in olive plants is needed for the breeding and domestication of a cold-resistant cultivar.

Post-translational modification is another cold adaptation in plants. Ubiquitination controls the abundance, activity, subcellular compartmentalization, and trafficking of target proteins (Linden and Callis, 2020). The ubiquitin-proteasome system consists of ubiquitin, ubiquitin-activating enzyme (E1), ubiquitin-conjugating enzyme (E2), ubiquitin ligase (E3), and intact 26S proteasome (Linden and Callis, 2020). E3 ubiquitin ligase is the component that recognizes and tethers polyubiquitins to the target proteins. Ubiquitination can attenuate or activate immune signaling in response to ambient change and pathogen invasion (Xu et al., 2021). Protein homeostasis is maintained by polyubiquitination to ensure proper immune responses (Kong et al., 2021). Polyubiquitin chains of various linkages confer unique functions to ubiquitins. The small peptide of ubiquitin presents several lysine residues that can be used to assemble a polyubiquitin chain by the subsequent sequential conjugation of new ubiquitins, the most prevalent being Lys48 and Lys63 (Denuc and Marfany, 2010). The attachment of a Lys48 ubiquitin chain causes the tagged molecule to degrade, whereas Lys63 polyubiquitin chains are involved in a wider range of functions, including DNA repair, endocytosis, or nuclear export (Denuc and Marfany, 2010). The external surroundings, such as Flg22, can alter the relative abundance of polyubiquitin linkages, which increase the percentages of Lys63 linkages (Ma et al., 2021). By comparison with monoubiquitination, polyubiquitination contributes more to the cold stress response in olive. EVM0030049, in the center of the subregulatory network, is polyubiquitin-like isoform X2. Its function demands further exploration to determine its interaction mechanism underlying upstream and downstream metabolism.

## Conclusion

**Figure 7:**
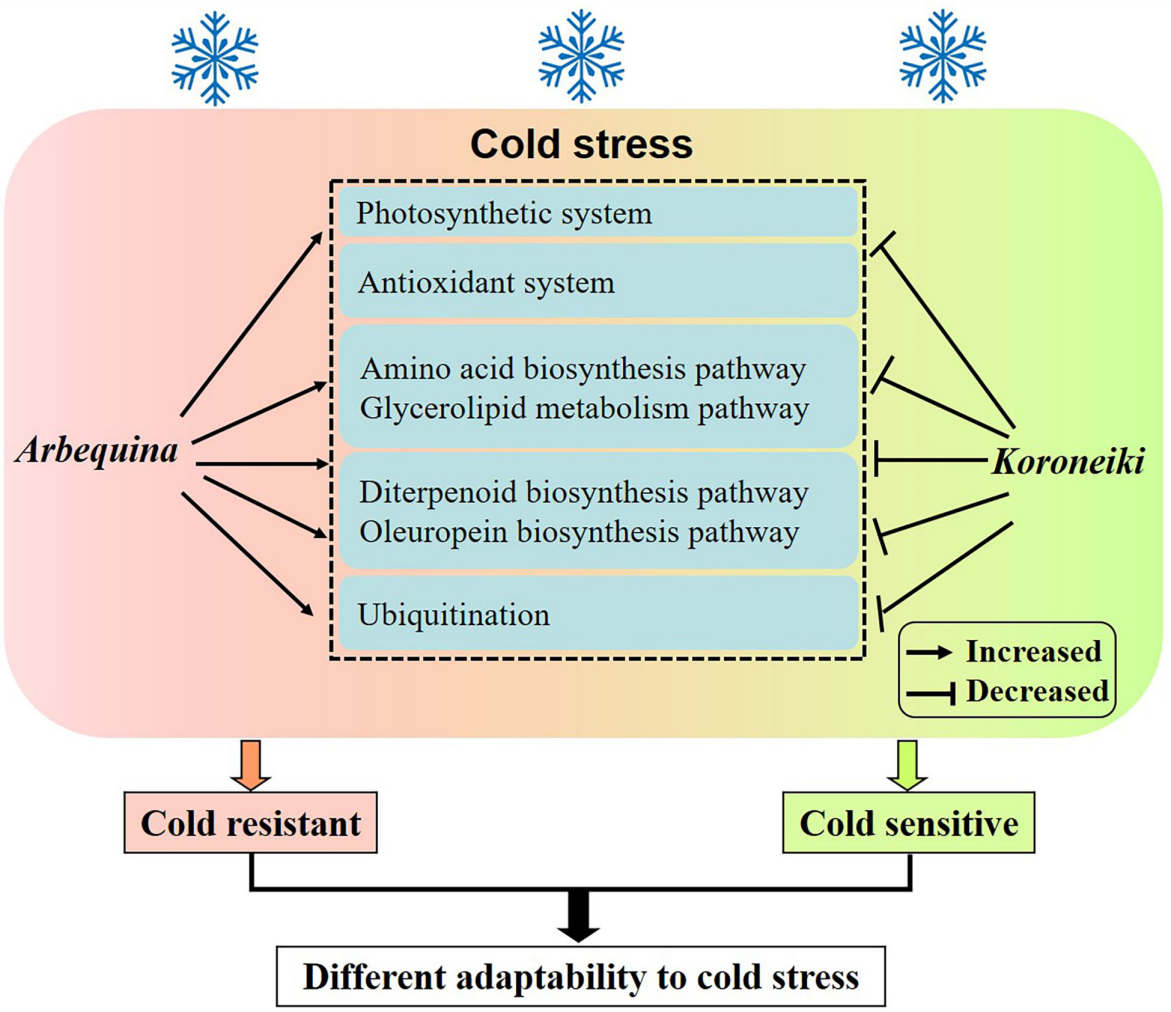
A proposed model for cold resistance in olive.

Cold-tolerant olive trees respond to cold stress through the amino acid biosynthesis, glycerolipid metabolism, diterpenoid biosynthesis, and oleuropein biosynthesis pathways, as well as through ubiquitination (**Figure 7**). For practical purposes, the breeding and selection of elite chilling-tolerant olive individuals should be undertaken after comprehensively evaluating these indicators.

## Data availability statement

The RNA-sequencing data in this study have been deposited in the NCBI Bioproject database under accession number (PRJNA864109).

## Author contributions

GS designed the experiments and revised the paper. CJ and WH performed the experiments and analyzed the data; CJ wrote the paper; HL, LC, EN, and SZ checked the data. All authors contributed to the article and approved the submitted version.
