# Supplementary material for: Alterations of phenotype, physiology, and functional substances reveal the chilling-tolerant mechanism in two common Olea Europaea cultivars
Source: Front Plant Sci. 2023 Feb 1;14:1046719. doi: 10.3389/fpls.2023.1046719 (PMC9930102; doi:10.3389/fpls.2023.1046719)
Supplement: Supplementary file 2 [file DataSheet_1.docx]

Supplementary figures

**FIGURE S1** **|** Classification of identified metabolites in leaves of olive.


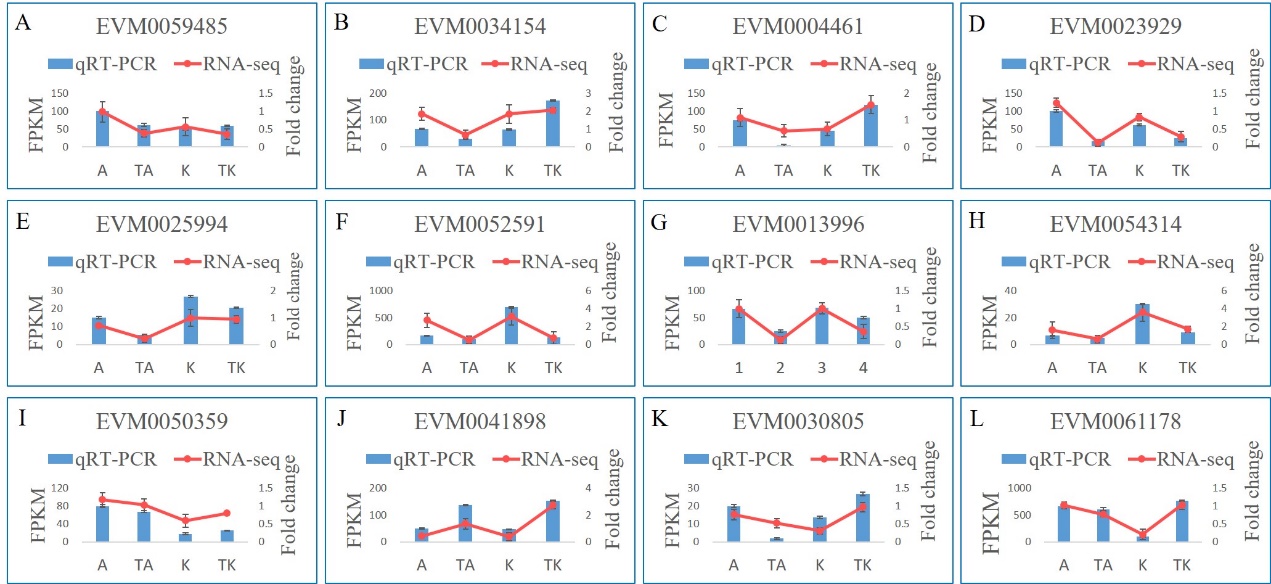


**FIGURE S2** **|** Verification of the relative gene expression levels by qRT-PCR analysis. **(A)** EVM0059485; **(B)** EVM0034154; **(C)** EVM0004461; **(D)** EVM0023929; **(E)** EVM0025994; **(F)** EVM0052591; **(G)** EVM0013996; **(H)** EVM0054314; **(I)** EVM0050359; **(J)** EVM0041898; **(K)** EVM0030805; **(L)** EVM0061178.
